# Machine Learning Integration of Bulk and Single-Cell RNA-Seq Data Reveals Cathepsin B as a Central PANoptosis Regulator in Influenza

**DOI:** 10.3390/ijms26178533

**Published:** 2025-09-02

**Authors:** Bin Liu, Lin Zhu, Caijuan Zhang, Dunfang Wang, Haifan Liu, Jianyao Liu, Jingwei Sun, Xue Feng, Weipeng Yang

**Affiliations:** 1 State Key Laboratory for Quality Ensurance and Sustainable Use of Dao-di Herbs, Institute of Chinese Materia Medica, China Academy of Chinese Medical Sciences, Beijing 100010, China; liubinzyy@163.com (B.L.); zhulin_948546@163.com (L.Z.); 18513031449@126.com (C.Z.); wdf122644@126.com (D.W.); 15530156755@163.com (H.L.); liujianyaojin@163.com (J.L.); 2 School of Traditional Chinese Medicine, Beijing University of Chinese Medicine, Beijing 100029, China; sunjingwei@bucm.edu.cn

**Keywords:** PANoptosis, influenza A virus, cathepsin B (CTSB), single-cell RNA sequencing, machine learning

## Abstract

Influenza A virus (IAV) infection triggers excessive activation of PANoptosis—a coordinated form of programmed cell death integrating pyroptosis, apoptosis, and necroptosis—which contributes to severe immunopathology and acute lung injury. However, the molecular regulators that drive PANoptosis during IAV infection remain poorly understood. In this study, we integrated bulk and single-cell RNA sequencing (scRNA-seq) datasets to dissect the cellular heterogeneity and transcriptional dynamics of PANoptosis in the influenza-infected lung. PANoptosis-related gene activity was quantified using the AUCell, ssGSEA, and AddModuleScore algorithms. Machine learning approaches, including Support Vector Machine (SVM), Random Forest (RF), and Least Absolute Shrinkage and Selection Operator (LASSO) regression, were employed to identify key regulatory genes. scRNA-seq analysis revealed that PANoptosis activity was primarily enriched in macrophages and neutrophils. Integration of transcriptomic and computational data identified cathepsin B (CTSB) as a central regulator of PANoptosis. In vivo validation in an IAV-infected mouse model confirmed elevated expression of PANoptosis markers and upregulation of CTSB. Mechanistically, CTSB may facilitate NLRP3 inflammasome activation and promote lysosomal dysfunction-associated inflammatory cell death. These findings identify CTSB as a critical mediatoCTSBr linking lysosomal integrity to innate immune-driven lung injury and suggest that targeting CTSB could represent a promising therapeutic strategy to alleviate influenza-associated immunopathology.

## 1. Introduction

Influenza is a viral respiratory infection that causes an acute febrile illness often accompanied by myalgia, headache, and cough. The emergence of influenza A viruses has triggered four global pandemics, each resulting in high mortality, significant public health threats, and substantial economic losses worldwide [1,2,3]. Since the 1918 “Spanish” influenza outbreak, highly pathogenic strains of influenza A virus have emerged unpredictably yet repeatedly, contributing to an estimated 50 million deaths over the past century [4]. Given the rapid variation in influenza viruses and the low rate of vaccine coverage, antiviral drug therapy remains the primary treatment for influenza. Anti-influenza drugs include M2 inhibitors (adamantanes, such as rimantadine and amantadine), neuraminidase inhibitors (NAIs, such as peramivir, zanamivir, and oseltamivir), and, more recently, the cap-dependent endonuclease inhibitor targeting the PA polymerase subunit (baloxavir) [5,6,7]. However, adverse drug reactions and the emergence of resistant viral strains have underscored the urgent need for the development of new, safe, and effective antiviral agents for both therapeutic and prophylactic purposes.

Following influenza virus infection, its invasion of alveolar epithelial cells initiates a coordinated cellular defense response. This triggers a cascade of pathogenic cellular events characterized by three distinct yet synergistic forms of cell death—pyroptosis, apoptosis, and necroptosis—collectively termed PANoptosis [8]. Pyroptotic cells undergo membrane pore formation with rapid osmotic lysis, apoptotic cells display controlled chromatin condensation and cytoplasmic shrinkage, while necroptotic cells exhibit cytoplasmic granulation with delayed membrane rupture [9,10]. While this orchestrated multi-modal cell death mechanism effectively eliminates virus-infected cells, its excessive activation induces pathological consequences, including increased alveolar epithelial barrier permeability, neutrophil infiltration, and cytokine storm formation. These perturbations collectively contribute to the progression of acute lung injury [11]. In clinical manifestations, this immunopathological cascade manifests as alveolar interstitial edema, impaired oxygenation function, and potential progression to respiratory failure in influenza patients [12]. The dynamic equilibrium of PANoptosis demonstrates critical regulatory significance in maintaining immune homeostasis post influenza infection.

Advances in sequencing technology have propelled the widespread adoption of single-cell RNA sequencing (scRNA-seq) in biomedical research. This pivotal technique enables detailed analysis of cellular heterogeneity and plays a critical role in investigating the pathogenesis of diseases [13]. Integrating machine learning algorithms with bioinformatic approaches facilitates the identification of novel diagnostic biomarkers [14,15]. Growing evidence suggests that PANoptosis plays a role in the pathogenesis of pulmonary diseases [16,17,18]. This study employed a multi-omics integration strategy, combining single-cell RNA sequencing (scRNA-seq) and bulk transcriptomic data, to systematically investigate hub genes orchestrating the pathological upregulation of PANoptosis in influenza-infected hosts. Machine learning algorithms were systematically applied to prioritize candidate genes, culminating in the identification of core regulator genes mechanistically linked to PANoptosis activation post-influenza. The biological relevance and predictive accuracy of these findings were further validated through preclinical murine models. This integrated approach not only delineates key molecular drivers of PANoptosis but also establishes a mechanistic foundation for developing targeted therapeutic interventions against influenza-associated immunopathology.

## 2. Results

### 2.1. Analysis of PANoptosis-Related Features in Influenza Using scRNA-Seq Datasets

**Figure 1:**
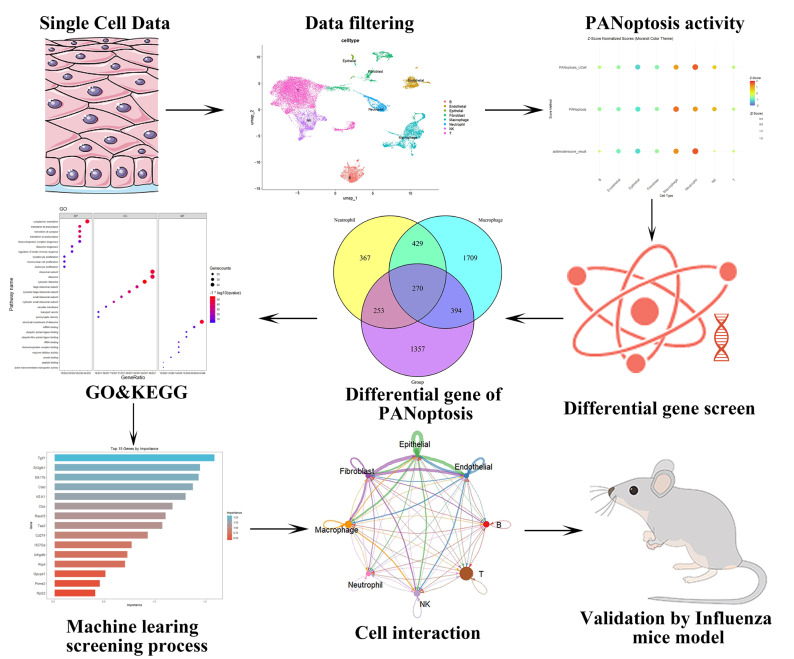
Schematic representation of the integrative transcriptomic analyses and machine learning workflow.

**Figure 2:**
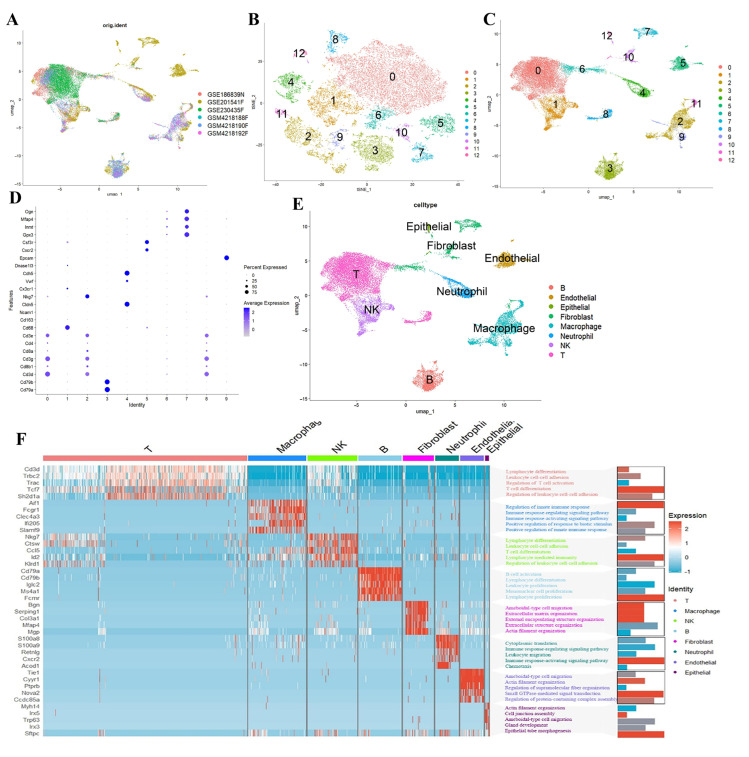
Integrative analysis of bulk GSVA and single-cell data processing. (**A**) PCA visualization demonstrated stable cellular distributions across samples with minimal batch effect susceptibility. (**B**) Clustering resolution was optimized to refine single-cell grouping. (**C**) UMAP analysis revealed systematic partitioning of cells into 12 distinct clusters. (**D**,**E**) Manual annotation using canonical marker genes categorized cells into 8 major types. (**F**): The relationship between the marker genes of the eight types of cells mentioned above, along with the relevant pathways enriched by GO analysis.

This study’s workflow is illustrated in Figure 1. We employed a scRNA-seq dataset to identify specific cell subsets demonstrating elevated PANoptosis activity, aiming to unravel the complexities of PANoptosis during influenza infection. Principal component analysis (PCA) revealed stable cellular distributions across all samples with minimal batch effect interference (Figure 2A). UMAP visualization demonstrated careful partitioning of cells into 12 distinct clusters (Figure 2B,C). Manual annotation using canonical marker genes successfully categorized these clusters into eight major cell types: T cells, endothelial cells, epithelial cells, fibroblasts, macrophages, neutrophils, natural killer (NK) cells, and B cells (Figure 2D,E). Gene Ontology (GO) analysis of pathway enrichment across these cell types provided deeper biological insights (Figure 2F).

**Figure 3:**
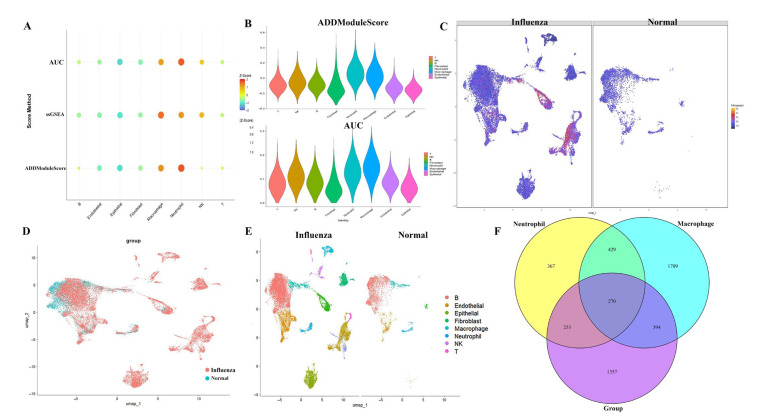
Single-cell PANoptosis dynamics during influenza infection. (**A**,**B**) Cross-algorithm validation using AUCell, ssGSEA, and AddModuleScore revealed elevated PANoptosis activity specifically in neutrophils and macrophages. (**C**–**E**) Spatiotemporal visualization of PANoptosis dynamics through UMAP projections comparing influenza-infected vs. control mononuclear cells. (**F**) Integrative Venn diagram analysis correlating PANoptosis-related pathways with DEGs identified through correlation analysis.

To investigate PANoptosis activity at the single-cell level post-influenza infection, PANoptosis scores for individual cells were calculated using the AUCell, ssGSEA, and AddModuleScore algorithms. These analyses revealed heterogeneity in PANoptosis activity across different cell types following H1N1 challenge. Neutrophils and macrophages exhibited the highest activity levels (Figure 3A,B). This elevated activity in neutrophils and macrophages was consistently visualized on UMAP plots (Figure 3C–E). We extracted genes specific to neutrophils and macrophages, resulting in 1319 genes for neutrophils and 2802 genes for macrophages. Differential expression analysis (DEG) was performed on all genes between the normal and influenza groups, yielding a total of 2274 DEG. A Venn diagram was constructed to intersect the DEG with the neutrophil and macrophage gene sets, revealing 270 genes (Figure 3F). These 270 genes represent differential genes in neutrophils and macrophages between the normal and influenza groups.

### 2.2. Results of GO Analysis and PPI Analysis

**Figure 4:**
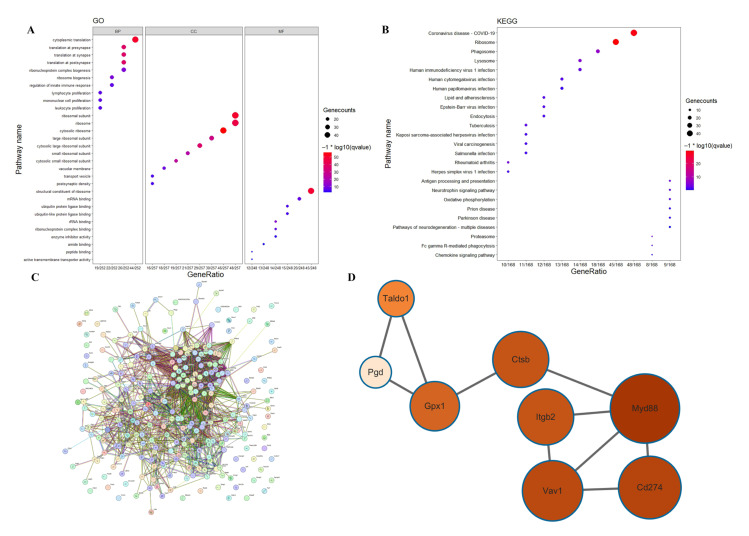
Results of GO analysis, KEGG analysis, and PPI analysis. (**A**): The results of GO analysis of the DEG genes. (**B**): The results of KEGG analysis of the DEG genes. (**C**): The results of PPI analysis of the DEG genes. (**D**): The results of MCODE analysis.

We conducted GO analysis to elucidate the relationships between the 270 positively regulated PANoptosis genes and their roles in various biological processes (Figure 4A). For instance, within the biological process (BP) category, the results indicate a focus on processes related to translation, biogenesis, immune response regulation, and cell proliferation, including in lymphocytes, mononuclear cells, and leukocytes. In terms of cellular component (CC), the analysis highlights components related to ribosomal subunits, ribosomes, transport vesicles, vacuolar membranes, and postsynaptic density. Regarding molecular function (MF), the results indicate a focus on activities related to ribosome structure, binding to mRNA, rRNA, ribonucleoprotein complexes, ubiquitin ligases, and various binding functions, such as amide, peptide, and active transmembrane transporter activity.

The KEGG pathways highlight interconnected mechanisms underlying infectious diseases, immune responses, and neurodegeneration. Viral carcinogenesis pathways (e.g., HPV, EBV, KSHV) and bacterial infections (Salmonella, Mycobacterium tuberculosis) intersect with host processes like endocytosis, phagocytosis, and antigen presentation. Dysregulated lipid metabolism and oxidative phosphorylation contribute to atherosclerosis and viral replication. Neurodegenerative pathways (Parkinson’s, prion diseases) and proteasomal dysfunction link to mitochondrial stress and neurotrophin signaling, while viral infections (HIV-1, HSV-1, HCMV) exploit host ribosomes and lysosomes, underscoring complex host–pathogen interactions in disease pathogenesis (Figure 4B). The results of PPI analysis revealed that these genes were closely interconnected and exhibited high functional correlation (Figure 4C). MCODE was employed to identify candidate hub genes from the protein–protein interaction (PPI) network of 270 differentially expressed genes (DEGs), including CTSB, Taldo1, Pgd, Gpx1, Itgb2, Myd88, Vav1, and Cd274 (Figure 4D).

### 2.3. Results of Machine Learning Algorithms

**Figure 5:**
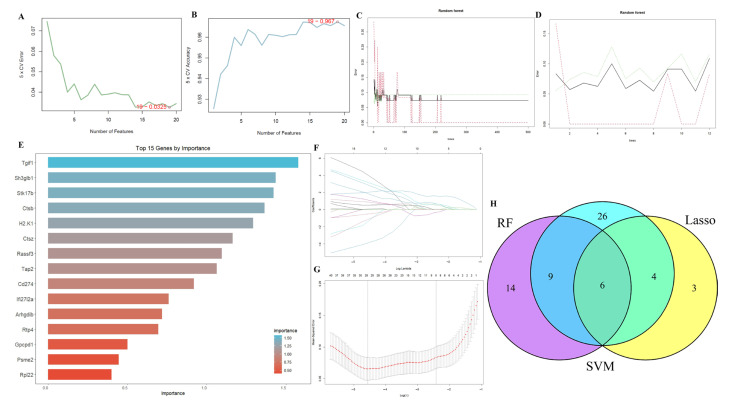
Machine learning algorithms evaluated the optimal feature genes. (**A**,**B**): SVM Classification Performance: 5-fold cross-validation error rate (%) and accuracy rate (%) as functions of feature set size. (**C**,**D**): Determination of optimal tree count in Random Forest analysis. (**E**): Top 15 gene features ranked by importance in Random Forest analysis. (**F**,**G**): Lasso variable screening process. (**H**): The results of Venn diagram of the above four machine learning algorithms.

Three machine learning algorithms were deployed to identify core feature genes from the initial 270 candidates. SVM analysis prioritized 45 key genes (Figure 5A,B), while the RF algorithm revealed 29 genes with non-zero importance scores (Figure 5C,D). LASSO regression further refined this list to 13 diagnostic markers for PANoptosis (Figure 5E–G). Cross-validating these results through algorithmic intersection, we ultimately identified six optimal feature genes: Rpl35a, Btg1, Gpcpd1, Psme2, Tra2b, and CTSB (Figure 5H). Notably, CTSB emerged as the sole overlapping gene when comparing this panel with previously identified hub genes, highlighting its potential as a critical PANoptosis regulator.

### 2.4. Comprehensive Validation of Core Feature Genes at Single-Cell Resolution and Analysis of Cellular Communication Networks

**Figure 6:**
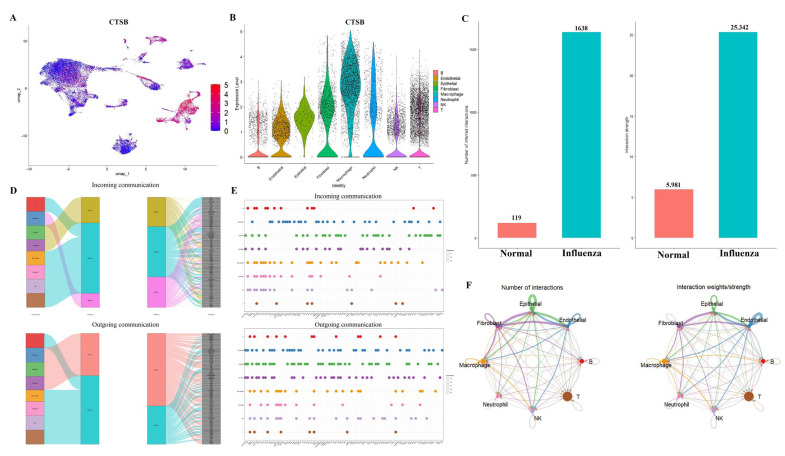
Validation of the optimal feature genes at the single-cell level and analyzing cell-cell interactions. (**A**): The results of UMAP indicated that CTSB was predominantly expressed in macrophages. (**B**): The results of violin plot for CTSB. (**C**): Intercellular communication quantity and intensity in control and influenza-infected cohorts. (**D**,**E**): The results of metabolic pathway analysis in monocytes. (**F**): The analysis of cellular communication revealed the magnitude and frequency of intercellular interactions among distinct cell types.

The results of UMAP demonstrated that CTSB exhibited high expression in macrophages (Figure 6A). The results of the violin plot for CTSB indicated elevated gene expression in macrophages and T cells (Figure 6B). Comparative analysis of cellular communication revealed a marked increase in both the number of interacting cells and the intensity of signaling in the influenza-infected group compared to the normal control group (Figure 6C,F). Figure 6D,E illustrates the outgoing and incoming pathways for each cell population, with the size of the circles representing the degree of contribution. The figure also illustrates ligand–receptor interactions between different cell types. Our analysis revealed that macrophages bind to other cells via the App–C74, Cd52–Siglecg, and Lgais9–Ighm receptor–ligand pairs.

### 2.5. Histopathological and Molecular Characterization of Influenza-Induced PANoptosis

**Figure 7:**
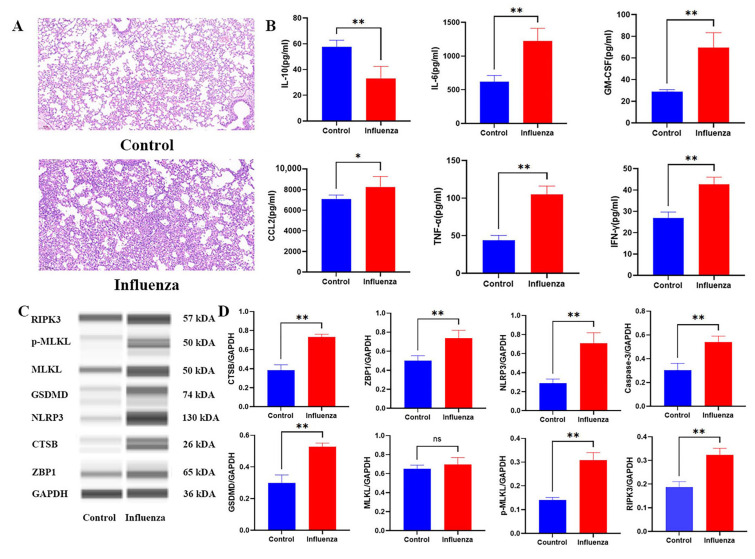
The results of experimental verification of influenza mice. (**A**): Histopathological examination via H&E staining revealed significant pathological alterations in pulmonary tissues of influenza-infected mice, with markedly evident inflammatory cell infiltration. Scale bars = 100 μm. *n* = 3 per cohort. (**B**): Assessment of pro-inflammatory cytokine levels (including CCL2, GM-CSF, IL-6, IFN-γ, and TNF-α) demonstrated a significant elevation in the influenza-infected group compared to controls. Conversely, the anti-inflammatory cytokine IL-10 exhibited a marked decrease. (*n* = 6/group). (**C**,**D**): The results of western blotting revealed that compared to the control group, the protein expression of Zbp1, CTSB, MLKL, p-MLKL, Caspase-3, RIPK3, NLRP3, and GSDMD increased post influenza (*n* = 3/group). For statistical significance we labeled **, * and ns to indicate *p*-values of <0.01, <0.05, >0.05, respectively.

To further validate the pathological features and molecular mechanisms underlying PANoptosis in influenza infection, we performed H&E staining, cytokine profiling, and Western blot analysis. Histological examination revealed severe alveolar damage and inflammatory infiltration in the lungs of infected mice, with significantly higher histopathological scores than the control group (Figure 7A). Concurrently, pro-inflammatory cytokines, including IL-10, CCL2, GM-CSF, IL-6, IFN-γ, and TNF-α, were markedly elevated (Figure 7B), suggesting a robust inflammatory response. Notably, Western blot analysis confirmed the upregulation of PANoptosis-associated proteins, including CTSB, MLKL, p-MLKL, Caspase-3, RIPK3, NLRP3, and GSDMD (Figure 7C,D). The increased expression of ZBP1 implies its potential role as an upstream sensor initiating PANoptosis signaling. These results collectively support the activation of PANoptosis as a key driver of immunopathology in influenza infection.

## 3. Discussion

Influenza A virus (IAV) has long presented a formidable challenge to global public health, with its high mutability and antigenic drift circumventing population immunity established through vaccination [19,20]. Despite advancements in antiviral therapies, such as neuraminidase inhibitors (oseltamivir) and PA endonuclease blockers (baloxavir marboxil), the persistent burden of severe pneumonia and acute respiratory distress syndrome (ARDS) underscores unmet clinical needs [21]. Beyond direct cytopathic effects of viral replication, accumulating evidence implicates dysregulated PANoptosis—a coordinated activation of pyroptosis, apoptosis, and necroptosis—as a central driver of immunopathology in influenza-associated lung injury [22]. During IAV infection, viral RNA sensors (e.g., ZBP1/DAI) recognize Z-form nucleic acids released from ruptured endosomes, triggering the assembly of the PANoptosome complex. This multi-protein platform recruits RIPK3, caspase-3, NLRP3, and MLKL to execute interlinked death pathways [23,24,25]. Critically, PANoptosis not only eliminates infected epithelial cells but also unleashes damage-associated molecular patterns (DAMPs), such as mitochondrial DNA (mtDNA) and high-mobility group box 1 (HMGB1), which hyperactivate alveolar macrophages through the TLR9 and RAGE receptors [26,27]. Monocytes/macrophages recruited to the lung microenvironment further amplify inflammation via TNF-α/IFN-γ synergy, culminating in a cytokine storm that disrupts endothelial barrier integrity [28,29]. These findings highlight PANoptosis as a conserved pathway bridging innate immunity and tissue damage across diverse diseases [30].

Our analysis revealed that macrophages and neutrophils displayed the highest PANoptosis activity, suggesting their significant contribution to immunopathology. Machine learning-driven integration of transcriptomic networks and PPI-MCODE analysis identified CTSB as the central hub gene, while simultaneously revealing other key regulators such as MYD88 and CD274 (PD-L1). Notably, MYD88, a key adaptor molecule for TLR and IL-1R signaling, promotes NF-κB activation and inflammasome priming, thereby indirectly amplifying pyroptosis through synergistic overactivation of the TNF-α and NLRP3 pathways [31,32]. CD274 (PD-L1) exerts immunomodulatory effects by binding to PD-1 on T cells, suppressing T cell activation and cytokine production. Dysregulation of PD-L1 in macrophages may impair antiviral immunity by inhibiting T cell-mediated viral clearance, potentially exacerbating chronic inflammation and tissue damage through indirect pathways [33,34]. This study ultimately pinpointed the optimal feature gene, CTSB, associated with heightened PANoptosis. Intriguingly, we discovered that CTSB was also significantly expressed in PANoptosis after H1N1. Several studies have proposed critical roles for immune cells and specific genes in PANoptosis regulation and H1N1 pathogenesis. Min Zheng et al. suggested that caspase-6 interacts with RIPK3 to enhance the RIPK3–ZBP1 interaction, promoting PANoptosome assembly, and demonstrated its requirement for alternative activation of alveolar macrophages during IAV infection [35]. Sk Mohiuddin Choudhury assessed the protein expression and compared the responses of immune and non-immune cells of human and mouse origin to canonical pyroptotic, apoptotic (staurosporine), necroptotic, and PANoptotic stimuli [36].

During influenza infection, PANoptosis, a collective term for apoptosis, necroptosis, and pyroptosis, plays a crucial role in modulating the host response. ZBP1 (Z-DNA binding protein 1), an interferon-inducible protein, acts as an innate sensor of influenza A virus (IAV) proteins, specifically the nucleoprotein (NP) and polymerase subunit PB1, triggering cell death and inflammatory responses via the RIPK1–RIPK3–Caspase-8 axis. ZBP1 deficiency protects mice from mortality during IAV infection by reducing inflammatory responses and epithelial damage, highlighting the importance of ZBP1 in the pathogenesis of IAV infection [24]. ZBP1-dependent cell death pathways, including apoptosis and necroptosis, contribute to the overall PANoptosis response, which balances viral clearance and host tissue damage. Furthermore, ZBP1 regulates NLRP3 inflammasome activation and the production of IL-1β and IL-18, which are protective during acute IAV infection. These findings underscore the complex interplay between innate immune sensing, cell death pathways, and inflammatory responses in shaping the outcome of influenza infection.

CTSB (Cathepsin B), a lysosomal cysteine protease, plays a pivotal role in intracellular protein degradation and homeostasis, participating in autophagy, apoptosis, and antigen presentation [37,38]. Dysregulated CTSB expression is implicated in cancers, where it enhances tumor invasion and metastasis by degrading extracellular matrix components, serving as a prognostic biomarker in breast cancer and gliomas [39]. Cathepsin-facilitated invasion of BMI1-high hepatocellular carcinoma cells drives bile duct tumor thrombi formation [40]. While CTSB’s multifaceted roles in health and disease are increasingly recognized, further research is needed to elucidate its tissue-specific functions and translational applications. The lysosomal membrane permeabilization (LMP) resulted in the release of CTSB. The released CTSB then activated the NLRP3 inflammasome, which in turn triggered pyroptosis. Therefore, CTSB acts as a key mediator linking the necroptosis pathway (via p-MLKL) to the pyroptosis pathway (via NLRP3), contributing to the overall PANoptosis process [41]. During IAV infection (0–24 hpi), CTSB exhibits temporally distinct roles facilitating pathogenesis. In the initial phase (0–6 hpi), CTSB promotes viral entry/replication and regulates apoptosis. Although non-essential for cellular entry, CTSB aids viral trafficking and hemagglutinin (HA) surface expression. Concurrently, CTSB secretion markedly increases by 6 hpi, correlating with inflammasome activation (CASP1 cleavage, IL-18 release) [42]. Progressing to the mid-phase (6–24 hpi), CTSB mediates inflammatory responses and initiates apoptosis. This involves lysosomal membrane permeabilization (LMP), cytosolic CTSB release, and subsequent caspase cascade activation, linking lysosomal dysfunction to programmed cell death. Thus, CTSB acts as a multifaceted regulator integrating viral replication, immune activation, and cell fate during early IAV infection [43].

The interplay between PANoptosis mediators and cytokine signaling networks further shapes the immunopathological landscape during influenza infection. Elevated levels of TNF-α, a pivotal pro-inflammatory cytokine, correlate with the ZBP1–RIPK1/3-dependent necroptosis and pyroptosis pathways, amplifying tissue inflammation and epithelial barrier disruption [44,45,46]. IL-6, a hallmark cytokine of acute viral infection, synergizes with TNF-α to amplify NLRP3 inflammasome activation, thereby inducing caspase-1-dependent pyroptosis through IL-1β and IL-18 maturation and contributing to neutrophil recruitment [47,48,49]. IL-10, a critical anti-inflammatory cytokine, exerts regulatory counteractions by attenuating RIPK1-mediated necroptosis and caspase-8-dependent apoptosis, thereby modulating excessive immune activation and mitigating immunopathology through its broad suppressive effects on pro-inflammatory cytokine networks [50,51,52]. MCP-1 (CCL2), a chemokine pivotal for monocyte recruitment and macrophage chemotaxis, is upregulated in inflammatory conditions in association with GM-CSF. GM-CSF not only sustains myeloid cell survival but also synergizes with MCP-1 to drive pro-inflammatory polarization of macrophages [53,54]. The combined upregulation of MCP-1 and GM-CSF promotes monocyte-derived macrophage infiltration into inflamed tissues, where GM-CSF further primes macrophages to enhance CTSB-mediated NLRP3 inflammasome activation, thereby amplifying inflammatory pyroptosis [55,56,57]. Notably, GM-CSF amplifies TNF-α and IL-6 synthesis via the autocrine/paracrine loops, generating a self-reinforcing inflammatory circuit that may synergize with PANoptosis-associated factors to escalate multicellular death and tissue injury [58,59]. These cytokine dynamics underscore the delicate equilibrium between viral containment and immune-mediated injury. Future studies interrogating spatiotemporal cytokine profiles in conjunction with cell death pathway activation could refine therapeutic strategies to preserve antiviral efficacy while minimizing bystander tissue damage.

The clinical translation of CTSB-targeted therapeutics is advancing. VBY-376, a small-molecule CTSB inhibitor for hepatic fibrosis/NASH, demonstrates robust preclinical efficacy, favorable preliminary human safety, and progress into Phase II trials [60]. Autophagy modulators chloroquine (CQ) and hydroxychloroquine (HCQ) act via lysosomal acidification disruption, inhibited autophagosome–lysosome fusion, and direct CTSB inhibition [61,62]. The novel lysosomal inhibitor Lys05 (structure-optimized from chloroquine, CQ) efficiently accumulates within lysosomes, exerts autophagy inhibition, and demonstrates single-agent antitumor activity in vivo [63]. Beyond oncology, CQ/HCQ exert anti-inflammatory effects through CTSB pathway modulation, showing potential in ischemic-reperfusion injury and autoimmune disorders [64,65]. Collectively, these diverse agents—spanning novel specific inhibitors to repurposed, multi-mechanism drugs—demonstrate the broad therapeutic potential of modulating the CTSB activity.

While this study advances our understanding of PANoptosis regulation in influenza pathogenesis, several limitations warrant attention. First, the translational relevance of findings in murine models to human infections remains unclear, necessitating validation in primate models or human lung organoids. Furthermore, we did not analyze the temporal dynamics of CTSB-mediated NLRP3 activation, and its crosstalk with other PANoptosis components was not fully elucidated in vivo, leaving causal relationships underexplored. Future work should address stage-specific interventions: early CTSB inhibition to curb immunopathology versus late-phase boosting to enhance viral clearance. The role of ZBP1 isoforms in human susceptibility to severe influenza remains unexplored, offering a precision medicine avenue. By bridging molecular mechanisms with clinical paradigms, such efforts could reposition PANoptosis modulators as adjuncts to antiviral therapies.

## 4. Materials and Methods

### 4.1. Source of Data

We accessed the GSE99192 bulk dataset from the Gene Expression Omnibus (GEO) database (https://www.ncbi.nlm.nih.gov/geo, accessed on 9 September 2024), which comprises lung and blood samples from 12 healthy mice and 43 influenza-infected mice. Additionally, we downloaded the scRNA-seq datasets—GSE186839, GSE201541, GSE230435, and GSE142047—from the GEO database for further analysis. Among these, GSE186839 serves as a control with a lung sample from a healthy mouse, while GSE201541 and GSE230435 each include a lung homogenate from an influenza-infected mouse, and GSE142047 contains lung homogenates from three influenza-infected mice.

We selected PANoptosis-related metabolic genes from the GeneCards database (https://www.genecards.org/, accessed on 9 September 2024), resulting in a total of 34 genes that were included in the analysis for this study.

### 4.2. scRNA-Seq Dataset Analysis

scRNA-seq data were initially converted into Seurat objects within the Seurat R framework. Cellular quality control was implemented with stringent filtering criteria: cells were retained only if they contained 200–7000 detected genes, exhibited mitochondrial gene expression below 20%, and demonstrated red blood cell gene expression under 3%. Dimensionality reduction was performed via principal component analysis (PCA) on highly variable genes, retaining the first 20 principal components (PCs) for downstream analysis. Cellular subpopulations were identified using the ‘FindClusters’ algorithm (resolution = 0.8), followed by visualization in two-dimensional uniform manifold approximation and projection (UMAP) space. Cluster-specific marker genes were subsequently defined by applying Seurat’s ‘FindAllMarkers’ function to compare transcriptomes against all other clusters. Annotation of clusters to established biological cell identities was performed via cross-referencing with canonical marker genes.

### 4.3. Evaluation of PANoptosis Activity

This study applied the AUCell [66], ssGSEA [67], and AddModuleScore [68] algorithms to assess PANoptosis activity at the single-cell level and calculate total PANoptosis activity. Correlation analysis was then performed to identify genes closely associated with PANoptosis activity. The ‘FindMarkers’ function was used to conduct differential gene expression (DEG) analysis to identify genes upregulated in PANoptosis. The genes identified through correlation analysis and DEG were subsequently examined in further analyses.

### 4.4. Enrichment Analysis

We performed gene ontology (GO) analysis to investigate the pathways and associated protein functions of these genes. To further validate the relevance of the genes to PANoptosis, we conducted protein–protein interaction (PPI) analysis to evaluate the interactions among these genes.

### 4.5. Machine Learning Algorithms for Identifying the Optimal Feature Genes

To pinpoint PANoptosis-related feature genes from the pre-filtered candidates, we implemented three distinct machine learning algorithms: Support Vector Machine (SVM) [69], Random Forest (RF) [70], and Least Absolute Shrinkage and Selection Operator (LASSO) [71]. Utilizing 5-fold cross-validation for parameter tuning and model optimization, we systematically applied each algorithm to identify core feature genes. This approach enhanced model robustness and mitigated overfitting. Genes consistently detected across all three analytical frameworks were ultimately defined as the key PANoptosis-associated feature genes.

### 4.6. Interactions Between Intercellular Communication and Transcription Factors

We integrated gene expression data using CellChat to evaluate variations in hypothesized intercellular communication modules, employing the default CellChatDB as the ligand–receptor database. Cell type-specific interactions were inferred by identifying overexpressed ligands or receptors within cell groups and detecting enhanced ligand–receptor interactions associated with this overexpression. Additionally, we utilized the R package Scenic (v1.3.0) to infer the activity of gene regulatory networks.

### 4.7. Animals and Experimental Design

Twenty-four specific pathogen-free (SPF) ICR mice (12 males and 12 females; 13–15 g) were acquired from SPF (Beijing, China) Biotechnology Co., Ltd. (Animal Production License No.: SCXK [Jing] 2023-0077). Mice were housed in an SPF facility under controlled conditions (temperature: 25 ± 2 °C; humidity: 60 ± 5%) with a 12 h light/12 h dark cycle. Irradiated rodent chow (Keao Xieli Feed Co., Ltd., Beijing, China) and sterile water were provided ad libitum. After 3-day acclimatization, animals were randomly allocated to two groups (*n* = 12/group, 6 males/6 females) via computer-generated sequence: (1) control and (2) influenza model.

For intranasal challenge, mice were anesthetized with diethyl ether (Sinopharm Chemical Reagent Co., Ltd., Shanghai, China) via induction chamber until loss of righting reflex (<5 min). Anesthetic depth was monitored by hind-paw withdrawal reflex. The influenza group received 50 μL A/H1N1 FM1 strain (diluted 1:640 in PBS; 35 μL virus suspension + 15 μL sterile PBS, pH 7.4), while controls received equivalent-volume 0.9% saline, administered as divided aliquots (25 μL/nostril) with 2 h intervals.

All procedures were approved by the Ethics Committee of the Institute of Chinese Materia Medica, China Academy of Chinese Medical Sciences (Approval No. 2024D022; 11 June 2024), and complied with Chinese Guidelines for Laboratory Animal Care (GB/T 35892-2018, https://openstd.samr.gov.cn/bzgk/gb/newGbInfo?hcno=9BA619057D5C13103622A10FF4BA5D14, accessed on 11 June 2024) and ARRIVE 2.0 guidelines.

### 4.8. Evaluation of Influenza Model in Mice

To characterize the influenza disease model, we performed quantitative cytokine profiling in lung tissues using Luminex multiplex technology and conducted histopathological evaluation via hematoxylin–eosin (H&E) staining across all experimental mouse cohorts. After weighing, the entire lungs were aseptically excised, rinsed twice with PBS, and blotted dry with filter paper. The lung tissues were harvested from mice and immediately fixed in 10% neutral-buffered formalin at room temperature for 24 h. Subsequently, the fixed tissues underwent dehydration, paraffin embedding, and sectioning into 4 μm-thick slices. Following standard histopathological procedures, the sections were stained with hematoxylin and eosin (H&E) to evaluate inflammatory responses and lymphocyte infiltration within the pulmonary architecture. Histological assessments were performed using light microscopy with examination of multiple randomly selected fields to comprehensively evaluate the distribution and severity of inflammatory cell infiltration. Precisely weighed pulmonary tissue samples were homogenized with ice-cold physiological saline at a tissue-to-saline ratio of 1:9 using a tissue homogenizer. The homogenate underwent centrifugation at 12,000× *g* for 15 min at 4 °C. The resultant supernatant was collected for subsequent analysis. The lyophilized standard was reconstituted in Universal Assay Buffer, thoroughly mixed, and maintained on ice to prepare the master standard solution. This solution underwent serial dilution using a two-fold dilution series to generate standard concentration points, which were then kept on ice. Subsequently, 50 μL of uniformly resuspended antibody-coupled magnetic beads was aliquoted into a 96-well microplate. The plate was positioned on a magnetic separation station, allowing for bead sedimentation and subsequent supernatant removal. Wells were rinsed twice with Wash Buffer. Following the addition of Universal Assay Buffer, 25 μL of prepared standards or samples was dispensed into corresponding wells. The plate was sealed with a specialized adhesive membrane and incubated for 30 min at ambient temperature under constant agitation to facilitate efficacious antigen–antibody interactions. Post-incubation, the plate was repositioned on the magnetic separation station for supernatant removal and subjected to three rigorous wash cycles with Wash Buffer. Subsequently, Streptavidin–Phycoerythrin (PE) conjugate was added to each well. After resealing, the plate underwent a secondary incubation period at room temperature with agitation. The sealing membrane was then removed, and sample quantification was performed using the Bio-Plex 200 system (Bio-Rad Laboratories, Inc., Hercules, CA, USA). Data analysis entailed standard curve generation via non-linear regression methodology, with analyte concentrations interpolated algorithmically.

### 4.9. Capillary Western Blotting

Characterization of PANoptosis was performed using an automated capillary electrophoresis western blotting system (WES™, ProteinSimple, San Jose, CA, USA) [72]. Protein lysates (1 mg/mL) were prepared by mixing 5.6 µL of the sample with 1.4 µL of fluorescent master mix and denatured at 95 °C for 5 min. The lysates, along with blocking reagent, primary antibodies (1:1000 dilution of anti-CTSB, anti-ZBP1, anti-GSDMD, anti-RIPK3, anti-*p*-RIPK3, anti-NLRP3, anti-MLKL, anti-p-MLKL, anti-Caspase-3 and anti-GAPDH; Abcam, Cambridge, UK), secondary HRP-conjugated antibodies, and chemiluminescent substrate, were loaded into the manufacturer’s microplate. The WES system automatically conducted electrophoretic separation and immunodetection using a 12–230 kDa separation module under default settings. Chemiluminescence was detected through an exposure series for optimal signal capture. Data analysis was conducted with Compass software (v6.3.0) (ProteinSimple, San Jose, CA, USA), producing electropherograms and virtual blot images to quantify the chemiluminescence signal for comparative purposes. GAPDH was used as the internal control for normalizing protein expression levels.

### 4.10. Data Analysis

All data were analyzed using SPSS 27.0 (SPSS Inc., Chicago, IL, USA). A one-way ANOVA procedure was used to examine the statistically significant differences in the data, and multiple comparisons were performed using Duncan’s method. Graphs were plotted using GraphPad Prism 8.0.2 (GraphPad Software, San Diego, CA, USA) for graphing, and the results of the tests are presented as means ± the standard deviations, with *p* < 0.05 indicating a significant difference and *p* < 0.01 indicating a highly significant difference.

## 5. Conclusions

This study integrates scRNA-seq and bulk RNA-seq with machine learning to identify cathepsin B (CTSB) as a pivotal regulator of PANoptosis in influenza A virus (IAV) infection. We demonstrate that PANoptosis is predominantly activated in macrophages and neutrophils during IAV challenge. Computational approaches (SVM, RF, and LASSO) prioritized CTSB among hub genes, which was validated in vivo by its upregulation and association with elevated PANoptosis markers (MLKL, caspase-3, NLRP3, and GSDMD) and cytokine release (IL-6, TNF-α). Mechanistically, CTSB promotes NLRP3 inflammasome assembly and lysosomal membrane permeabilization, amplifying inflammatory cell death. These findings establish CTSB as a critical link between lysosomal dysfunction and immunopathological lung injury, highlighting its potential as a therapeutic target to mitigate influenza-associated PANoptosis and acute respiratory distress.

## Data Availability

The raw data supporting the conclusions of this article will be made available by the authors on request.

## Abbreviations

The following abbreviations are used in this manuscript:

AUCell	Area Under the Recovery Curve algorithm
BP	Biological Process (in Gene Ontology analysis)
CC	Cellular Component (in Gene Ontology analysis)
CTSB	Cathepsin B
DEGs	Differentially Expressed Genes
GO	Gene Ontology
GSVA	Gene Set Variation Analysis
GSDMD	Gasdermin D
IAV	Influenza A Virus
IFN-γ	Interferon gamma
IL-	Interleukin (e.g., IL-1β, IL-6, IL-10)
LASSO	Least Absolute Shrinkage and Selection Operator
LMP	Lysosomal Membrane Permeabilization
MCODE	Molecular Complex Detection algorithm
MF	Molecular Function (in Gene Ontology analysis)
MLKL	Mixed Lineage Kinase Domain Like Pseudokinase
NF-κB	Nuclear Factor Kappa-Light-Chain-Enhancer of Activated B Cells
NK cells	Natural Killer cells
NLRP3	NLR Family Pyrin Domain Containing 3
NASH	Non-Alcoholic Steatohepatitis
PAMP	Pathogen-Associated Molecular Pattern
PCA	Principal Component Analysis
PPI	Protein-Protein Interaction
RAGE	Receptor for Advanced Glycation Endproducts
RF	Random Forest
RIPK1/3	Receptor-Interacting Serine/Threonine-Protein Kinase 1/3
scRNA-seq	Single-Cell RNA Sequencing
SVM	Support Vector Machine
ssGSEA	Single-sample Gene Set Enrichment Analysis
SPF	Specific Pathogen-Free
TLR	Toll-Like Receptor
TNF-α	Tumor Necrosis Factor alpha
UMAP	Uniform Manifold Approximation and Projection
ZBP1	Z-DNA Binding Protein 1
